# *Staphylococcus aureus* and *Pseudomonas aeruginosa* Express and Secrete Human Surfactant Proteins

**DOI:** 10.1371/journal.pone.0053705

**Published:** 2013-01-22

**Authors:** Lars Bräuer, Martin Schicht, Dieter Worlitzsch, Tobias Bensel, R. Gary Sawers, Friedrich Paulsen

**Affiliations:** 1 Department of Anatomy II, Friedrich-Alexander-University Erlangen-Nürnberg, Erlangen, Germany; 2 Department of Hygiene of the medical faculty of the Martin Luther University of Halle-Wittenberg, Halle, Germany; 3 Institute for Biology/Microbiology, Martin Luther University of Halle-Wittenberg, Halle, Germany; The Scripps Research Institute and Sorrento Therapeutics, Inc., United States of America

## Abstract

Surfactant proteins (SP), originally known from human lung surfactant, are essential to proper respiratory function in that they lower the surface tension of the alveoli. They are also important components of the innate immune system. The functional significance of these proteins is currently reflected by a very large and growing number of publications. The objective goal of this study was to elucidate whether *Staphylococcus aureus* and *Pseudomonas aeruginosa* is able to express surfactant proteins. 10 different strains of *S. aureus* and *P. aeruginosa* were analyzed by means of RT-PCR, Western blot analysis, ELISA, immunofluorescence microscopy and immunoelectron microscopy. The unexpected and surprising finding revealed in this study is that different strains of *S. aureus* and *P. aeruginosa* express and secrete proteins that react with currently commercially available antibodies to known human surfactant proteins. Our results strongly suggest that the bacteria are either able to express ‘human-like’ surfactant proteins on their own or that commercially available primers and antibodies to human surfactant proteins detect identical bacterial proteins and genes. The results may reflect the existence of a new group of bacterial surfactant proteins and DNA currently lacking in the relevant sequence and structure databases. At any rate, our knowledge of human surfactant proteins obtained from immunological and molecular biological studies may have been falsified by the presence of bacterial proteins and DNA and therefore requires critical reassessment.

## Introduction

Four surfactant proteins have been described to date (SP-A, SP-B, SP-C and SP-D), that were first detected in the lung [1], [2], [3], [4]. The proteins differ considerably in structure, function and biochemical properties. SP-A and SP-D are representatives of the C-type lectins that have immunological functions in non-specific and specific immune defense; SP-B and SP-C are among the smallest and most hydrophobic proteins of all. Their physicochemical properties enable them to reduce the surface tension of biological interfaces and contribute to the adsorption of phospholipids at the air-liquid interface [5], [6]. The first description of SP-B and SP-C in organic extracts was by Phizackerley as early as 1979. Characterization and purification of these proteins proved very difficult due to their high level of hydrophobicity and low molecular weight [7]. The immunological and surfactant properties of surfactant proteins give them an enormous pathophysiological significance. Loss of these proteins leads to increased alveolar surface tension, causing alveolar atelectasis in pulmonary respiration, thus hindering gas exchanges and weakening the alveolar immune defense [8]. Up to now there are thousands of publications dealing with surfactant proteins. For comprehensive review confer [9], [10], [11], [12], [13].

A vast number of investigations have demonstrated that bacterial cell wall components (especially those of *Staphylococcus aureus* and *Pseudomonas aeruginosa*) exert an immense influence on the synthesis of surfactant proteins [14], [15], [16], [17]. Stimulation experiments with bacterial supernatants of *S. aureus* and *P. aeruginosa* have therefore been used for some time *in vitro* to simulate bacterial infections in commonly used cell culture models [18], [19], [20]. It must also be said that not a single study to date has been able to confirm or disprove whether the microorganisms or their supernatants show reactivity to the commercially available antibodies and ELISA systems. In our own preliminary work, commercial SP-specific antibodies were tested against the bacterial supernatants, with the astonishing result that antibody reactions were confirmed: a result of great significance and relevance for hundreds of scientific studies. The object of the present paper was a more detailed analysis of these surprising results. Answers were also sought to the question of whether *S. aureus* and *P. aeruginosa* – both opportunistic human pathogens and “problem germs” in pulmonary infections – might themselves be capable of producing surfactant proteins or other comparable proteins.

## Materials and Methods

### Used bacteria and their cultivation

The bacteria strains used are listed in Table 1. The bacteria were cultured in LB liquid medium overnight for 10–12 h at 37°C in a shaker incubator (approx. 220 rpm).

**Table 1:** Bacterial strains used in this study.

Organism	Strain collection	Common name	Origin, other information
***Staphylococcus aureus***	ATCC 35556	SA 113	Institute for Hygiene Martin Luther University, Derived from NCTC 8325, restriction deficient
	ATCC 12228	SA N315	Robert Koch Institute Berlin, Patient isolated 1982, hospital-acquired methicillin-resistant
	NCTC 8325		Robert Koch Institute Berlin, inducible erythromycin resistance
		SA 8	Institute for Hygiene Martin Luther University, Patient Isolate
		SA 16	Institute for Hygiene Martin Luther University, Patient Isolate
***Pseudomonas aeruginosa***		PA 01	Institute for Hygiene Martin Luther University
	ATCC 14442		Institute for Hygiene Martin Luther University
	ATCC 27853		Institute for Hygiene Martin Luther University
		PA 154	Institute for Hygiene Martin Luther University, Environmental Isolate
		PA 12	Institute for Hygiene Martin Luther University, patient Isolate
***Escherichia coli***	ATCC 35218		Institute for Hygiene Martin Luther University
		BL21	Institute for Hygiene Martin Luther University
***Pyrococcus furiosus***	DSM 3638		Leibnitz-institute DSMZ-German Collection of Microorganisms and Cell Cultures

### Bacterial RNA/DNA preparation

A purification system from Zymo Research (ZR Bacterial RNA Mini Prep.) was used to isolate the RNA from the bacteria based on the manufacturer's protocol. The required cell pellet was first obtained from a 5 ml bacterial culture by means of centrifuging. To isolate genomic DNA, the bacteria were proliferated in a shaker culture overnight at 37°C in LB (Luria Bertani) liquid medium. 5 ml of this culture were then centrifuged for 5 min at 13,000 rpm and room temperature (RT). The pellet was resuspended in 100 µl 10 mM Tris+25% sucrose, pH 7.5. 15 µl 0.5 M EDTA, pH 8, were also added. Lysis was achieved by using ultrasonic and adding 10 µl of lysostaphin (2 mg/ml) and incubation for 20 min at 37°C. 375 µl of TE buffer, 225 µl 10% SDS and 20 µl proteinase K (10 mg/ml) were then added and the mixture was incubated for 30 min at 55°C. To separate the plasmid DNA from the chromosomal DNA, 50 µl 5 M sodium perchlorate solution was added, the mixture was swirled briefly and a mixture of chloroform and isoamyl alcohol (24∶1) was added. After several inversions the proteins were initially precipitated by constant shaking (1 h). Following a centrifugation step (10 min at 13,000 rpm) the DNA was finally precipitated from the supernatant with a double volume of 100% ethanol. This was followed by centrifugation at RT for 10 min and 13,000 rpm. The DNA pellet was then washed with 70% ethanol and dried at RT for approx. 1 h. It was then assimilated in 50 µl distilled H_2_O and stored at 4°C.

### PCR

For conventional PCR we used conditions as previously described by us [21] with the primers given table 2. Bp values of the amplified fragments were compared with gene bank data [22] For verification and comparison, lung tissue obtained from body donors was used as a reference. PCR products were also confirmed by BigDye sequencing (Applied Biosystems, Foster City, CA). A ß-actin PCR was performed to proof the bacterial DNA/RNA for contamination with human DNA/RNA. GyrA PCR was used as positive control for presence of bacterial DNA/RNA within *S. aureus* and *P. aeruginosa*. Bacterial 16S PCR was used as positive control for presence of bacterial DNA/RNA in *Pyrococcus furiosus*.

**Table 2:** Sequences of the primers used for detection of surfactant proteins (A; B; C; D), RT-PCR analysis.

Primer	Sense primer, 5′→3′	Antisense primer, 5′→3′	bp	°C
SP-A	GAT GGG CAG TGG AAT GAC AGG	GGG AAT GAA GTG GCT AAG GGT G	212	56
SP-B	CAC CAT GTT CCC CAT TCC TCT	TCA TCC ATG GAG CAC CGG AGG ACG	239	60
	CAA ACG GCA TCT GTA TGC AC	CGG AGA GAT CCT GTG TGT GA	194	52
SP-C	TCA TCG TCG TGG TGA TGG TG	ATG GAG AAG GTG GCA GTG GTA A	110	55
	CTG GTT ACC ACT GCC ACC TT	TCA AGA CTG GGG ATG CTC TC	142	57
SP-D	TGC TGC TCT TCC TCC TCT CTG C	GGG CGT TGT TCT GTG GGA GTA G	95	55
	AGG AGC AAA GGG AGA AAG TGG G	CAG CTG TGC CTC CGT AAA TGG	199	55
β-Actin	CAA GAG ATG GCC ACG GCT GCT	TCC TTC TGC ATC CTG TCG GCA	275	60
Gyrase A	TGT GCT TTA TGC CAT GAG CGA	TCC ACC GAA CCG AAG TTG C	220	58
16S RNA	CGG GGC GCA GCA GGC GCG AA	ACG GGC GGT GTG TGC AA	1000	60

### Antibodies

Antibodies (displayed in table 3) were used for Western blot analysis as well as for immunohistochemical investigations as specified by the manufacturer.

**Table 3:** Molecular weights of the surfactant proteins and specific antibodies used for their detection in Western blot analysis and Immunofluorecence Micorscopy.

Protein	Molecular weight (kDa)	Antibody	Company, Catalog number
SP-A	28–36; 66	Mouse monoclonal anti human SP-A	Millipore; MAB3270
SP-A	28–36; 66	Rabbit polyclonal anti human SP-A	Santa Cruz; sc-7700
SP-B	8; 18; 40	Mouse monoclonal anti human SP-B	Acris; DM3204
SP-B	8; 18; 40	Rabbit polyclonal anti human SP-B	Abcam; AB40786
SP-B	8; 18; 40	Mouse monoclonal anti human SP-B	Millipore; MAB3276
SP-C	4–6; 6–12; 21; 26	Rabbit polyclonal anti SP-C human	Chemicon; AB3786
SP-D	43	Mouse monoclonal anti human SP-D	Acris; BM4083
SP-D	43	Mouse monoclonal anti human SP-D	Acris; BM4005
SP-D	43	Rabbit polyclonal anti human SP-D	Santa Cruz; sc-7708

### Isolation of bacterial proteins

For Western blot analysis bacterial proteins were extracted from *Staphylococcus aureus* and *Pseudomonas aeruginosa*. The bacteria were grown in LB-medium for 12 hours at 37°C and afterwards harvested by centrifugation at 4°C at 13.000 rpm for 15 min. The supernatant referring to LB-medium containing secreted bacterial proteins as well as bacterial surface constituents like LPS or PGN was separated and stored at −20°C. The bacterial pellet was resuspended in 300 µl 1%-Triton X-100 buffer with 1% lysozyme. After ultrasonic treatment and incubation on ice the cocktail was centrifuged for 30 min at 13.000 rpm for separation of cell-wall and insoluble constituents (pellet) from the bacterial cytosol (supernatant). The concentration of the total proteins was determined for each sample using a protein assay kit (BioRad Laboratories, Richmond,VA).

### Western blot analysis

For Western blot analysis bacterial proteins were isolated and measured with a protein assay based on the Bradford dye-binding procedure (BioRad, Hercules, CA). Total protein (30 µg) was then analyzed by Western blot. Proteins were resolved by reducing 15% SDS-polyacrylamide gel electrophoresis, electrophoretically transferred at room temperature for 2 h at 0.8 mA/cm2 onto 0.1 µm pore size nitrocellulose membranes and fixed with 0.2% glutaraldehyde in phosphate-buffered saline for 30 min. Bands were detected with primary antibodies to SP-A (1∶500) SP-B (1∶250), SP-C (1∶500), SP-D (1∶500) and secondary antibodies (anti-rabbit/anti-mouse IgG, respectively, conjugated to horseradish peroxidase, 1∶5.000) applying chemiluminescence (ECL- Plus; Amersham-Pharmacia, Uppsala, Sweden). Human lung was used as control. The molecular weights of the detected protein bands were estimated using standard proteins (Prestained Protein Ladder, Fermentas, St. Leon-Rot, Germany) ranging from 11 to 170 kDa.

### Immunoelectron microscopy

Bacteria were grown in LB medium and fixed with 4% PFA. After several washes with PBS, the bacteria were treated with the primary antibody 1∶50–100. Incubation was done overnight at 4°C. Then, bacteria were washed repeatedly with PBS, followed by incubation with gold-labeled antibodies for 2 hours. The bacteria were washed with PBS followed by incubation with 2.5% glutaraldehyde. The samples were incubated with 0.5% osmium tetroxide and after washout with PBS. Then they were treated with silver enhancement. The bacteria were washed 2 times with distilled water and mixed with 4% low-melt agarose until the agarose solidified. The agarose block containing the homogenously distributed bacteria was stored for 2 days in 70% ethanol.

After dehydration in graded concentration of ethanol, the bacteria block was incubated twice 1∶1 in a 100% ethanol: acetone mixture and once in 100% acetone. Finally the bacteria block was infiltrated with increasing concentrations of Epon with the following acetone-Epon-mixtures: 1∶3 Epon, 2∶3 acetone and 2∶3 Epon, 1∶3 acetone and Epon 100% without acetone. The embedded block was polymerized at 60°C for 24 h and 90°C for 48 h.

### Process for cutting ultrathin sections

Semithin sections of embedded bacteria were examined. The 50 nm sections were trimmed with a diamond knife. The sections were taken up from water with 200-mesh copper, dried and counterstained with aqueous uranyl-acetate and lead citrate to increase the contrast for transmission electron microscopy. The grids were examined with a transmission electron microscope (Zeiss 900).

### Immunofluorecence micorscopy

For immunostaining of the bacteria we used conditions as previously described by [23].

### Enzyme-linked immunosorbent assay (ELISA)

The ELISA analysis was performed using kits and the regarding protocols from USCN Life Science Inc. Wuhan. By comparing with the standard series and the determined values for antigen concentration (protein concentration), each sample was calculated in ng/mg.

### Statistical analysis

The data used were the mean+SE (standard error of the mean (SEM)) of the samples tested for expression. Statistical significance was calculated with the two-tailed t-test; analyzed (GraphPad Software, San Diego, CA InStat statistical software). p<0.05 indicated significance.

## Results

### Detection of surfactant protein genes within bacterial DNA and RNA

Both genomic DNA and the RNA from different samples of *S. aureus and P. aeruginosa* were investigated using PCR and RT-PCR for presence of the genetic information for surfactant proteins. To exclude potential contamination with human foreign DNA, all analyses were accompanied by a control PCR with specific primers for detection of human β-actin, which showed no amplification product. Bacterial gyrase A served as the positive control. Lung tissue was used as the internal product control; *E. coli* (see Methods) was only used as an internal product control for the gyrase A PCR. Sterile water was used as the internal negative control (no-template control).

**Figure 1:**
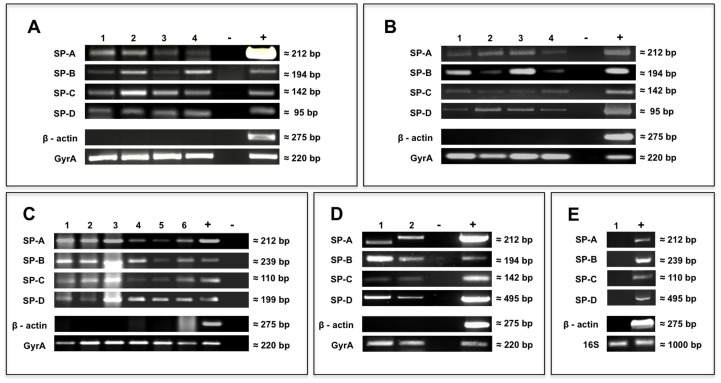
PCR and RT-PCR analysis of bacterial strains. **A**) RT–PCR analysis for transcripts encoding surfactant proteins (A, B, C, D) from *S. aureus* displaying the samples from different strains; 1. SA 113 [ATCC 35556] aerobe, 2. SA 113 [ATCC 35556] anaerobe, 3. SA N315 [ATCC 12228] aerobe, 4. SA [NCTC 8325] aerobe. **B**) RT–PCR analysis for transcripts encoding surfactant proteins (A, B, C, D) from *P. aeruginosa* displaying the samples from different strains; 1. PA 01 aerobe, 2. PA 01 anaerobe, 3. PA [ATCC 14442] aerobe, 4. PA [ATCC 27853] aerobe. **C**) PCR analysis for genomic DNA from *S. aureus* and *P. aeruginosa* displaying the samples from different strains; 1. PA 01 Laboratory strain; 2. PA 154 Environmental isolate, 3. PA (12) Patient isolate, 4. SA 113 [ATCC 35556] Laboratory strain, 5. SA (8) Patient isolate, 6. SA (16) Patient isolate. **D**) PCR analysis of bacterial DNA from (1) *E. coli* BL21 (no plasmid) and from (2) *E. coli* [ATCC 35218] (plasmid). In each case a (RT-)PCR using ß-actin (human), GyrA (bacterial) was performed to include/exclude bacterial/human contamination. (+) indicates the internal positive control for the PCR experiments. E) PCR analysis of bacterial DNA from (1) *Pyrococcus furiosus* that serves as negative control.

For each analyzed mRNA sample of *S. aureus* (Figure 1A) and *P. aeruginosa* (Figure 1B), the specific PCR product for the specific surfactant protein was detected (SP-A = 212 bp, SP-B = 194 bp, SP-C = 142 bp, SP-D = 95 bp). Figure 1C summarizes the result of the bacterial genome analysis. The specific amplification products were confirmed in all samples tested (SP-A = 212 bp, SP-B = 239 bp, SP-C = 110 bp, SP-D = 199 bp). All of the levels measured corresponded to the expected primer-specific product sizes and were verified by sequencing, resulting in identity levels of 91–100%. The negative control and β-actin showed no amplification products. Gyrase A was positive in all samples used except the negative control. 16S PCR was positive in samples of *Pyrococcus furiosus* (cf. figure 1E).

### Analysis of surfactant protein genes within bacterial plasmids

Detection of the genes raised the question of whether the genes were encoded in the genome itself or at the plasmid level. The analysis was carried out with the chemocompetent *E. coli* BL21 and in comparison with a laboratory strain (cf. table 1).

Figure 1D illustrates the plasmid analysis results. The specific PCR products for the corresponding surfactant proteins were detected in all samples (SP-A = 212 bp, SP-B = 194 bp, SP-C = 142 bp, SP-D = 495 bp). All of the levels measured corresponded to the expected primer-specific product sizes and were verified by sequencing, resulting in identity levels of 91–100%. The negative control and β-actin showed no amplification products. Gyrase A was positive in all samples used except the negative control.

### Detection of bacterial surfactant proteins by means of immunofluorescence and immunogold-labelling

Since Western Blot determined specific antibody reactions, immunofluorescence and immunogold-labelled cultured bacteria (*S. aureus* and *P. aeruginosa*) were analyzed using electron microscopy.

**Figure 2:**
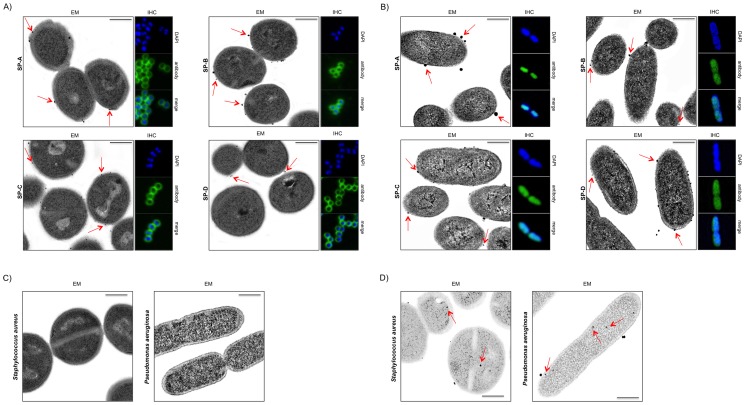
Multi imaging of *S. aureus* and *P. aeruginosa* by means of immunofluorescence and immunogold electron microscopy. Detection of SP-A, -B, -C and –D by means of immunofluorescence (IHC) and immunogold electron microscopy (EM) for *S. aureus* (A) and *P. aeruginosa* (B). The electron microscopically images reveal positive antibody reactivity indicated by the black dots surrounding the bacterial cells. Some of them are marked by using red arrows. In both bacterial strains the antibody reactivity is recognizable mainly on the surface of the microorganisms but also in the cytosol at least to some extend (cf. (D)). Figure D shows cytosolic labeling within the 50 nm ultrathin sections after decreasing the intensity of the background. In case of *S. aureus* (A) this result is additionally enhanced by the immunofluorescence images (green staining). As proof for unspecific binding of the used antibodies, bacteria were treated and incubated with secondary antibody only (cf. (C)). Neither *S. aureus* nor *P. aeruginosa* revealed any reactivity with the antibody.

All of the surfactant proteins investigated were detected based on the corresponding antibody reactions at the outer cell wall of *S. aureus* and *P. aeruginosa* (cf. Figure 2 (A) and 2 (B)). The green staining marks the positive antibody reaction for the specific surfactant protein on the outer bacterial cell wall. Superimposing the two images revealed membrane association of the detected proteins. Immunogold electron microscopy of ultrathin sections of the bacteria (50 nm) further supports this result. Circular gold particles (red arrows in Figures 2(A) and 2(B)) indicate positive antibody binding to the outer cell wall of the bacteria. To exclude unspecific binding of antibodies to *S. aureus* and *P. aeruginosa* bacteria were incubated with the secondary antibody only. In no case immunogold-labeling of the 50 nm ultrathin sections could be observed (cf. Figure 2(C)).

### Detection of bacterial surfactant proteins by means of Western Blot

**Figure 3:**
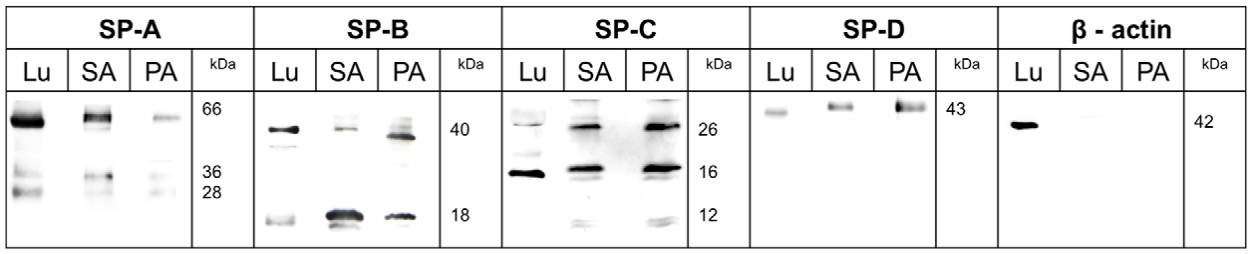
Western Blot analysis of bacterial surfactant proteins using monoclonal antibodies originated against human SP-A, -B, -C and -D. Western blot analysis after SDS gel electrophoresis of bacterial proteins from *S. aureus* (SA) and *P. aeruginosa* (PA). After cultivation the proteins were extracted from the bacteria. All investigated samples show distinct bands at the known molecular weights for human surfactant proteins. Lung tissue (Lu) was used as positive control and shows in each case the expected bands for the respective surfactant proteins. ß-actin, a human protein absent in bacteria, was used as a negative control and shows no band.

To facilitate immune detection of the surfactant proteins A, B, C and D in protein samples from *S. aureus* and *P. aeruginosa*, they were isolated from the bacteria and analyzed using Western Blot. Lung tissue served as the control. For each investigation we first checked the supernatant of the bacterial broth (referring to LB-medium containing proteins and substances secreted by the bacteria) for reactivity with human surfactant protein antibodies. In further steps we lysed the bacterial pellets into membrane/cell wall as well as cytosolic fractions and performed Western blot analysis with both fractions (cf. Materials and Methods). Each fraction revealed reactivity with the human surfactant protein antibodies. Figure 3 exemplarily illustrates detection of the surfactant proteins in the bacterial supernatants based on distinct bands. Within the bacterial supernatants the strongest immunological signal could be detected. However, all other fractions revealed similar results albeit demonstrating weaker signals within the Western blot experiments. These data have been omitted for clarity reasons and are not shown. For SP-A, including the control tissue (lung), a specific band was detected at 66 kDa. For SP-B, in comparison with the lung tissue, bands were detected at 40 kDa and 18 kDa in both bacteria samples. Protein detection of SP-C reveals several bands compared to the lung tissue control. The first band is at 12 kDa and is visible only for the bacteria. The second band at 16 kDa is present in all samples. The third band, at 26 kDa, is also visible in all samples. The test for SP-D reveals a detectable band at 43 kDa. This corresponds to the known protein size for SP-D. All obtained distinct bands match with the published molecular weights of the different posttranslationally modified or processed human surfactant proteins which can be detected using the regarding antibodies (cf. Table 3).

### Quantification of bacterial surfactant proteins by means of ELISA under different growth conditions

Proliferation trials followed by ELISA were to clarify whether the bacteria regulate the production of the surfactant proteins under stress. To this end, the facultative anaerobic bacterial *S. aureus* and *P. aeruginosa* were at first cultured for 48 hours under anaerobic and aerobic conditions, after which their proteins were isolated and quantified using ELISA. Table 4 shows the corresponding concentrations of surfactant proteins in ng/mg of total protein after 48 h of growth.

**Table 4:** Protein concentration in ng/mg total protein.

Bacterium and condition	Protein concentration mean value [ng/mg total protein]							
	SP-A		SP-B		SP-C		SP-D	
*Staphylococcus aureus* aerobic	0.9	*	132	*	7.0	*	100	*
*Staphylococcus aureus* anaerobic	2.0		390		26		320	
*Pseudomonas aeruginosa* aerobic	0.3		4.9		0.5		0.1	
*Pseudomonas aeruginosa* anaerobic	0.4		35		1.0		0.3	

**Figure 4:**
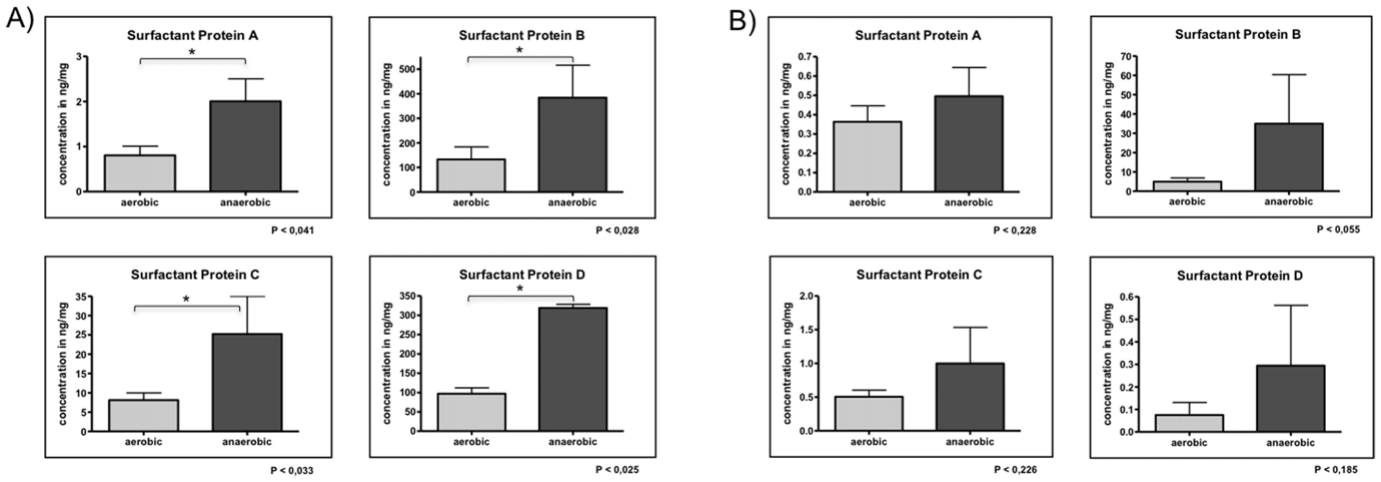
ELISA quantification of surfactant proteins A, B, C and D in *S. aureus* (A) and *P. aeruginosa* (B). ELISA quantification of SP-A, -B, -C and –D in *S. aureus* (A) and *P. aeruginosa* (B) after cultivation using different media conditions (aerobic and anaerobic). For *S. aureus* (A) the concentration of each surfactant proteins is significantly increased in case of anaerobic cultivation (significance in p is shown in the figure for each surfactant protein. *P. aeruginosa* also reveals a rise in protein concentration after anaerobic cultivation, but this is not significant.

Table 4 and Figure 4a show that the concentration of the investigated surfactant proteins increases significantly under anaerobic conditions.

Table 4 and Figure 4b shows results for *P. aeruginosa*. They present a similar picture, whereby however the amounts of surfactant proteins produced are smaller. Nonetheless, an increase in proteins expression could be observed, although this was not significant in the case of *P. aeruginosa*.

## Discussion

As related in the introduction, immunological and rheological functions are ascribed to the surfactant proteins. Above all the surfactant proteins SP-A and SP-D participate in numerous processes involved in the non-specific immune defense [24]. They are capable of binding and neutralizing both gram-negative and gram-positive bacteria by acting as so-called opsonins to represent part of the immune response, thus resulting in bacterial aggregation and facilitating phagocytosis [25], [26]. Both SP-A and SP-D, mediated by calcium ions, bind to lipopolysaccharides (LPS) of the bacteria [27]. SP-D reacts with the oligosaccharide of the LPS, whereas SP-A interacts with protein A [28]. An effect on pathogenic microorganisms, in particular *P. aeruginosa*, was also demonstrated for the smaller surfactant protein B [29]. In view of the many publications on the immunological significance of the surfactant proteins, this can actually be assumed to be confirmed in principle [30]. This makes the observations made here all the more astonishing. The literature has not described comparable results to date and the presence of SPs is theoretically excluded since they have potent antibacterial properties as described. The analysis of the genomic bacterial DNA showed specific bands for all surfactant proteins tested. This result is completely unexpected since a comparison of the primers with the available bacterial sequence databases did not turn up any correspondence with the bacterial genome, beside which the presence of surfactant proteins was not expected. To exclude human contamination at the genomic level, analyses were performed with the human-specific protein ß-actin, with negative results in the various microorganism samples tested. In view of the fact that the bacteria also use RNA as translation replicates, RNA detection testing was also done. Here as well, the results for *S. aureus* and *P. aeruginosa* show specific PCR products for the known human surfactant proteins. The specific controls confirm the data obtained and exclude contamination. Sequencing of the bands showed agreement with the corresponding human DNA sequence of 91 to 100 percent. The difference could be explained by sequential variants in the human genome such as also occur in pigs and cattle. A sequence comparison with the genomes of *S. aureus* and *P. aeruginosa* revealed no agreement.

The question is then how to explain the fact that, with contamination excluded, SP-specific PCR products are found at the mRNA and DNA levels, whereby the corresponding sequences do not occur in the bacterial genome. Current scientific knowledge considers the genomes of *S. aureus* and *P. aeruginosa* completely decoded. However, a couple of investigations demonstrate that there are still white spots within the genome of microorganisms. In this context Xue et al. recently demonstrated the presence of diverse genes in isolates of *S. aureus* resulting from horizontal gene transfer [31]. Furthermore, recent work using the latest techniques on other microorganisms (e.g. *Helicobacter pylori*) have revealed large numbers of new RNAs demonstrating that there are still white spots within the genome of microorganisms [32]. For instance, a new regulatory 6S-RNA was detected for the first time in *Helicobacter pylori*. This could suggest that, despite complete decoding of a genome, new methods and targeted search mechanisms may facilitate identification of new genes, e.g. also non-bacteria-specific ones. Handford also postulate a ubiquitous role of such proteins, which could also hold for the surfactant proteins [33]. This assumption is supported by the fact of detection of the surfactant proteins in many different human tissues [18], [34], [35], [36].

The fact that detection of surfactant proteins at the RNA level is not strain-specific was demonstrated by use of 3 different staphylococcus and pseudomonas laboratory strains (see Table 1, Methods). All of the laboratory strains are, however, human isolates, so that gene transfer between host and bacterium cannot be excluded. In this context we tested the DNA of *P. furiosus* for the presence of the known surfactant protein genes and could not detect any amplification product (cf figure 1E). *P. furiosus* can be assumed as a bacterium that never had relationship to the human organism and therefore gene transfer can be excluded. This result is additional evidence that *S. aureus* and *P. aeruginosa* might have integrated the DNA of the surfactant proteins to their own genome.

Normally, gene segments from mammals are rarely established in the genomes of microorganisms, since these pathogens possess numerous mechanisms, for example the CRISPR/Cas system [37] that protects them against foreign DNA. However, a recent report involving *Neisseria gonorrhoeae* describes how this pathogen has integrated an active human gene (LINE, L1) in its genome [38]. The mechanism of integration was not clarified. The authors assume, in view of the obligatory human pathogenic habitus of this organism, a constant contact, and thus constant interaction, with the nucleic acids of the host. These analyses assume an efficient system (horizontal gene transfer) for uptake of DNA by pathogenic microorganisms. A comparable uptake or assimilation of surfactant genes or gene segments could be assumed for *S. aureus* and *P. aeruginosa* as opportunistic human pathogenic microorganisms.

The assumption that the genes could be encoded on an unknown plasmid was also excluded. Commercial *E. coli* strains were used that no longer contain any plasmids. A comparison (with and without plasmid) detected specific PCR fragments in both strains. We therefore assume that the surfactant proteins are determined at the genomic level. This was also determined to be the case in *S. aureus* (not shown).

To confirm the molecular biological results, tests were carried out based on the relevant protein biochemistry. Western blot analysis using the commercial anti-surfactant protein antibodies detected specific bands. The results show specific bands compared to the positive control (human lung). For SP-A (28, 36 and 66 kDa), SP-B (18 and 40 kDa) and SP–C (12, 16 and 26 kDa) several bands were detected. For SP-D a classic protein size of 43 kDa was clearly determined. It is known from the literature that these surfactant proteins form oligomers of various orders of magnitude [39], [40], [41], [42]. The sizes also vary among the different tissues. For instance for SP-B 8 kDa have been described in the lung, over 25 kDa in the Clara cells and up to 35 kDa in the ocular system [43], [44]. In the case of SP-C, the protein mass varies from 26 kDa [45] to 21 kDa [44] and 16 kDa/7 kDa [46]. To exclude unspecific binding of the antibodies to the bacterial proteins we performed Western blot analyses using the secondary antibody only. To enhance and specify this investigation we additionally performed Western blot analysis with an antibody against human ß-actin (cf. Figure 3). In no case antibody reactivity was obtained. Therefore, unspecific binding can be excluded. Especially with regard to *S. aureus* this investigation is important because of the ability of S. aureus to express protein A which can lead to unspecific binding of immunoglobulins [47].

To specify and enhance these results we performed immunogold-labeled transmission electron microscopy using ultrathin sections of the bacteria (cf. figure 2). All investigated samples show specific and distinct reaction of the human surfactant protein antibodies illustrated by the black dots (representing the gold particles linked to the secondary antibodies) and the red arrows at the bacterial surface (cf. figure 2). Also in this investigation we incubated the 50 nm sections of the bacteria with the secondary antibodies only and did not find any unspecific binding (cf. Figure 2(C)). Results obtained performing immunofluorescence microscopy additionally support these findings (cf. figure 2).

The finding that protein biochemistry-based analysis of the bacteria cultures using classic anti-surfactant protein antibodies detects specific bands is both surprising and of considerable significance. Considering the fact that many cell culture experiments and in vivo studies on the effect of surfactant proteins have used bacteria cultures of different species and that human sample material can be colonized with bacteria, the results obtained here challenge the results of all of these investigations. All of the results obtained and publicized in this way, including our own work, must be critically reviewed and reanalyzed in view of the present findings. The question is raised as to whether the increased concentration of surfactant proteins detected with bacterial colonization [18], [20], [29], [48], [49], [50] is due to the presence of bacterial surfactant of the bacteria *S. aureus* and/or *P. aeruginosa and maybe also of other bacteria*. Clarification of this question will certainly be interesting and may change the way science sees the surfactant proteins.

Explanations of why bacteria need surfactant proteins must remain speculative at present. The fact is that bacteria can adapt rapidly to a host so as to avoid any selection. This adaptive capacity was demonstrated with five newly sequenced *Bartonellea* species (isolates from various mammals) [51]. The authors concluded that secretion systems obtained by means of horizontal gene transfer facilitate occupancy of various niches. In relation to the surfactant proteins investigated in the present study, this might supply a possible explanation for detection of the proteins. Uptake and integration into the bacterial genome would result in development of a selective advantage. In this scenario, the surface regulatory proteins B and C could simplify bacterial movement or even penetration into epithelial layers. In a summary article, Harshey describes the different modes of bacterial locomotion. It becomes apparent that the principle involved is often clear, but the participating proteins are unknown [52]. It is difficult to explain the presence of the immunoregulatory surfactant proteins A and D in the bacteria, particularly as various studies have demonstrated convincingly that SP-A and SP-D result in agglutination and opsonization of the bacteria [25], [26].

On the other hand, the flagellum of *P. aeruginosa* protects the microorganism from the effect of surfactant protein A and facilitates cell membrane permeation [53]. Lemos et al. (2011) demonstrated that SP-A and SP-D are not obligatory components in the immune response in mice [50]. This shows that the immunoregulatory properties can be sidestepped or play a subordinate role in the immune response. Therefore, the production of SP-A and SP-D-like proteins in the microorganisms investigated could have a “camouflaging” purpose to prevent opsonization processes or agglutination, or to enhance surface-regulatory processes.

Immunohistochemical and electron microscopy studies of *S. aureus* and *P. aeruginosa* have shown that these bacteria carry the proteins SP-A, B, C and D on their surface. External presentation is particularly important for the surface-regulatory properties, since the proteins mediate like an anchor between phospholipid layers and the aqueous phase [54]. It is known in this context that SP-A enhances the surface-regulatory properties of SP-B by way of interaction with SP-B [55]. This would make a “non-immunoregulatory” property in microorganisms plausible. Using electron microscopy, it was conclusively demonstrated that the commercially available antibodies bind to bacterial surfactant proteins present on the cell surface of the microorganisms.

ELISA experiments (cf. Figures 4A and 4B) were used to determine whether the proteins could also be regulated under certain conditions, e.g. oxidative stress. Bacteria were cultured under anaerobic and aerobic conditions. Both *S. aureus* and *P. aeruginosa* – facultative anaerobes – produce significantly larger concentrations of surfactant proteins under anaerobic conditions than under aerobic conditions. It is notable that the concentration of SP-B is many times that of SP-A, C and D for both microorganisms and that, all told, *P. aeruginosa* appears to produce less surfactant protein. This effect is presumably due to the investigative method applied here, since *P. aeruginosa* possesses a thick alginate shell that is lost in the sample preparation process. Since the surfactant proteins are detected on the surface of the microorganisms, these would thus also be lost. Stress factors influence with biofilming of *S. aureus* [56]. In this context it can be assumed that increased concentration of the bacterial surfactant proteins would, on the one hand, simplify the mobility of the microorganisms in the biofilm and, on the other hand, would also simplify accretion and agglutination of the bacteria, facilitating development of an optimized microclimate. In cystic fibrosis (CF) in particular, a frequent inherited metabolic disease in humans, formation of thick mucus and the related development of anaerobic conditions play a special role [57]. Various scientific studies describe a 50% increase in SP-A concentration in the sputum of CF patients [58], [59]. In this connection, it should be considered whether the SP-A increase might also be due to an increase in bacterial colonization in the mucus of CF patients and not, as postulated, solely to increased protein expression of SP-A.
